# Nanocluster Evolution in D9 Austenitic Steel under Neutron and Proton Irradiation

**DOI:** 10.3390/ma16134852

**Published:** 2023-07-06

**Authors:** Suraj Venkateshwaran Mullurkara, Akshara Bejawada, Amrita Sen, Cheng Sun, Mukesh Bachhav, Janelle P. Wharry

**Affiliations:** 1 School of Materials Engineering, Purdue University, West Lafayette, IN 47907, USA; 2 Department of Metallurgical and Materials Engineering, Indian Institute of Technology Madras, Chennai 600036, India; 3 Department of Mechanical Engineering & Materials Science, University of Pittsburgh, Pittsburgh, PA 15260, USA; 4 Intel Corporation, Hillsboro, OR 97124, USA; 5 Idaho National Laboratory, Idaho Falls, ID 83415, USA; cheng.sun@inl.gov (C.S.); mukesh.bachhav@inl.gov (M.B.)

**Keywords:** austenitic stainless steel, neutron irradiation, proton irradiation, nanoclusters, atom probe tomography

## Abstract

Austenitic stainless steel D9 is a candidate for Generation IV nuclear reactor structural materials due to its enhanced irradiation tolerance and high-temperature creep strength compared to conventional 300-series stainless steels. But, like other austenitic steels, D9 is susceptible to irradiation-induced clustering of Ni and Si, the mechanism for which is not well understood. This study utilizes atom probe tomography (APT) to characterize the chemistry and morphology of Ni–Si nanoclusters in D9 following neutron or proton irradiation to doses ranging from 5–9 displacements per atom (dpa) and temperatures ranging from 430–683 °C. Nanoclusters form only after neutron irradiation and exhibit classical coarsening with increasing dose and temperature. The nanoclusters have Ni_3_Si stoichiometry in a Ni core–Si shell structure. This core–shell structure provides insight into a potentially unique nucleation and growth mechanism—nanocluster cores may nucleate through local, spinodal-like compositional fluctuations in Ni, with subsequent growth driven by rapid Si diffusion. This study underscores how APT can shed light on an unusual irradiation-induced nanocluster nucleation mechanism active in the ubiquitous class of austenitic stainless steels.

## 1. Introduction

Austenitic stainless steels (SS) such as 304 SS and 316 SS are the most ubiquitous structural alloys in light water reactor (LWR) nuclear power plants [1,2]. But these alloys are susceptible to irradiation hardening and embrittlement due to the formation of voids, dislocation loops, and stacking fault tetrahedra [3,4,5,6,7,8,9,10]. These alloys also exhibit poor creep resistance, especially at elevated temperatures as would be required in advanced Generation IV nuclear reactors such as supercritical water-cooled reactors (SCWR) and sodium-cooled fast reactors (SFR) [11,12,13,14]. Consequently, 316 SS has been modified with a higher Ni/Cr ratio and higher Si and Ti concentrations for enhanced radiation tolerance [15,16,17,18,19] and high-temperature creep strength [20]. This modified material, known as alloy D9, has since been included in several neutron irradiation campaigns at the Fast Flux Test Facility (FFTF) [17,18,19,21], High Flux Isotope Reactor (HFIR) [15,22], Experimental Breeder Reactor-II (EBR-II) [16,23,24], and Advanced Test Reactor (ATR) [25].

The FFTF experiments focused on the irradiation-induced mechanical property evolution of D9. Over irradiation temperatures of 390–457 °C, fracture toughness degrades with increasing dose from ~45 dpa to ~135 dpa (corresponding to neutron fluences 9–27 × 10^22^ n/cm^2^) [18]. More generally, though, irradiation temperatures over the range of 395–686 °C have relatively little influence on post-irradiation mechanical properties, including reduction in ductility [21]. Meanwhile, the irradiation experiments in HFIR and EBR-II focus more on the irradiated microstructure evolution of D9. Maziasz [15,22] specifically studied void swelling of various process heats of D9 and found that swelling is suppressed at irradiation temperatures below 500 °C, even at doses as high as 34–57 dpa. Void incubation appears to give way to steady-state swelling when G-phase precipitation begins [16]. This kinetic relationship between voids and G-phases is due to the role of P and Si [22] in stabilizing fine, high-density precipitates, thus extending the void incubation time [15,23]. The sizes of both voids and G-phase precipitates increase with increasing irradiation temperature from 427 to 593 °C [23].

Despite these aforementioned irradiation effects studies on alloy D9, the irradiated microstructure and mechanical properties had not been directly related until Chen and colleagues [26] conducted nanoindentation and transmission electron microscopy (TEM) characterization of several ATR-irradiated D9 specimens. They observed conventional irradiation hardening behaviors as a function of dose. The hardening is directly related to the Frank loops, cavities, and irregular Ni–Si nanoclusters through the Orowan dispersed barrier hardening model (although cavities are ultrafine and have a relatively trivial contribution to overall hardening). However, the highest degree of hardening, which occurs at the highest irradiation dose, also occurs at the highest irradiation temperature. This is somewhat unusual, since the extent of hardening typically decreases with temperature. Due to the limited number of specimens, the role of temperature and dose could not be deconvoluted. However, the authors theorize that the Ni–Si nanoclusters may be able to explain the hardening behaviors.

A unique aspect of the irradiated microstructure that remains to be fully explored is this Ni–Si nanoclustering. Although Chen et al. [26] characterized these nanoclusters, their study notes specific gaps in the understanding of the chemistry and morphologies of these nanoclusters, which they believe will influence the mechanical behaviors and irradiation hardening. Chen and colleagues [26] note that they characterized the nanoclusters using TEM with energy dispersive X-ray spectroscopy (EDS), but, given the ~few nm resolution of TEM, and the through-thickness nature of EDS composition measurements, greater accuracy of the Ni–Si nanocluster compositions is warranted. Hence, the use of atom probe tomography (APT) will complement Chen’s work by providing three-dimensional, atomic resolution reconstruction of irradiation-induced nanoclusters in alloy D9.

The present study accesses the identical neutron-irradiated specimens of D9 previously characterized by Chen et al. [26]. The as-received archival material from the original D9 ingot is studied as a control specimen, and a piece of this ingot is also irradiated with protons to a dose and temperature that fall within the range of doses and temperatures of the neutron irradiations studied. APT characterization of all specimens reveals a fine dispersion of Ni–Si nanoclusters in the neutron-irradiated specimens. The role of irradiation dose and temperature on nanocluster morphology is discussed. The use of APT provides unique insight into the structure of nanoclusters, which can shed light on the mechanisms of Ni–Si nanocluster nucleation in irradiated austenitic steels.

## 2. Materials and Methods

### 2.1. Alloy Preparation and Irradiation

An ingot of Alloy D9 was obtained from Oak Ridge National Laboratory (Oak Ridge, TN, USA). The alloy composition is provided in Table 1 using APT and compared to scanning electron microscopy (SEM) EDS measurements from ref. [26] and the nominal composition [25]. While all composition measurements are in generally good agreement, especially for the major alloying species, APT is sensitive to minor elements with low concentrations, but is more susceptible to local heterogeneities. The D9 specimens were machined into discs of 3 mm diameter and ~500 µm thickness using electrical discharge machining (EDM). The disc faces were polished using SiC paper through P4000 grit to a thickness of ~250 µm.

Discs were loaded into capsules for neutron irradiation in the Advanced Test Reactor (ATR) at Idaho National Laboratory (INL) (Idaho Falls, ID, USA). The capsules were irradiated in the ATR East Flux Trap, which has a maximum fast flux of ~9.7 × 10^13^ n/cm^2^-s (E > 1 MeV), during cycles 143A through 150B; the specimen loading configuration is described in ref. [25]. Three D9 neutron-irradiated specimens were selected for this study, as shown in Table 2. The specimen geometries were 3 mm discs of ~200 µm thickness, so the neutrons can be assumed to deliver a homogeneous fluence throughout the specimen volume. These specimens accumulated total neutron fluences of 1.65 × 10^22^, 2.69 × 10^22^, and 3.07 × 10^22^ neutrons/cm^2^, corresponding to damage doses of 5, 8, and 9 displacements per atom (dpa), respectively. The irradiation temperatures were determined to be 448, 430, and 683 °C, respectively [27], based on ABAQUS calculations with input from reactor instrumentation and SiC melt wires, following methods described in ref. [28] with temperature uncertainties of approximately ±20 °C.

A proton irradiation was also conducted to conditions falling within the dose and temperature range bounded by the neutron irradiations, as shown in Table 2. For proton irradiation, a specimen from the same ingot of alloy D9 was cut into a 2 mm × 2 mm × 20 mm matchstick using EDM. The surface to be irradiated was mechanically polished through P4000 grit SiC paper. Subsequently, the specimen was electropolished in a 10% perchloric acid + 90% methanol solution maintained at −33 ± 3 °C with a Pt mesh electrode. The specimen was included in an irradiation stage with several other specimens of various steels, the irradiation results from which have been reported in refs. [29,30,31,32]. The proton–target interaction was calculated using SRIM 2013 in Quick Kinchin–Pease mode, and the dose of 7 dpa was determined at half the depth to the damage peak, or ~10 µm below the irradiated surface, as described in ref. [30]. The irradiation temperature was calibrated using thermocouples spot-welded to the specimen surfaces, and the temperature was tracked throughout the irradiation using a non-contact 2D infrared thermal pyrometer. The reader is referred to ref. [29] for comprehensive details about the proton irradiation experiment setup, control, and monitoring.

### 2.2. Atom Probe Tomography

APT needles were fabricated using established focused ion beam (FIB) milling procedures on a Thermo Fisher Scientific Quanta 3D FEG dual-beam SEM/FIB (Waltham, MA, USA) at the Center for Advanced Energy Studies (CAES) [33]. APT needle fabrication targeted grain interiors, aiming to avoid grain boundaries or near-boundary regions with potential denuded zones. A ~200 nm Pt protective layer was deposited onto the identified regions of interest, then trench cuts were made adjacent to the Pt layer. Using conventional under-cutting and extraction procedures, a lamella was lifted out and welded onto Si posts on an APT microtip array coupon. The microtips were subsequently milled using an annular pattern with sequentially decreasing FIB beam currents. Final beam energies of 5 keV and 2 keV were used to sharpen each needle to a diameter of <100 nm, while minimizing FIB damage on needle surfaces. In the proton-irradiated specimens, a larger wedge lift-out was extracted, such that the sharpened tips of the needles would be situated ~1 µm below the original irradiated surface, which would correspond to an actual irradiation dose of ~6 dpa.

The needles were tested in a CAMECA (Madison, WI, USA) Local Electrode Atom Probe (LEAP) 5000X HR, also at CAES, operating in laser pulse mode. The LEAP run parameters for each needle are summarized in Table 3; across all needles, the laser energy ranged from 0.06–0.14 NJ, pulse rate ranged from 200–250 kHz, specimen base temperature ranged from 39.5–60.5 K, and detection rate ranged from 0.5–1%. APT data was reconstructed using the proprietary CAMECA Integrated Visualization and Analysis Software (IVAS) version 3.8.4. A minimum of two needles were run from each specimen condition, and a minimum of ~1,000,000 ions were collected from each needle in order for the data set to be considered statistically sound. During reconstruction, the ranged Ni ions were determined using the peaks at both 29 and 30 Da in the time-of-flight mass spectrum. Although the 29 Da peak overlaps with Fe, this peak accounts for >68% of all Ni ions and only 0.3% of all Fe ions, based on natural isotopic abundances [34]. Thus, despite the higher bulk concentration of Fe than Ni in the D9 alloy, only a relatively negligible amount of Fe ions would be ranged as Ni ions in the 29 Da peak.

Cluster analysis was conducted within IVAS using the maximum separation method [35] for Si ions. The maximum allowable distance between solute atoms in a cluster, *d_max_*, and the minimum number of ions that must satisfy the *d_max_* condition to be considered a cluster, *N_min_*, were varied for each needle following recommendations from refs. [36,37,38,39]. The values of *d_max_* and *N_min_* were consistent within each irradiation condition and varied slightly across irradiation conditions, as shown in Table 3. Cluster analysis provided compositional and dimensional information for each cluster, and the total number of clusters identified per needle. The average nanocluster size was determined from the Guinier diameter, *D_G_*, as [40]:

(1) $$D_{G}=2\sqrt{\frac{5}{3}}R_{g}$$

where *R_g_* is the overall radius of gyration, which is estimated from the gyration radii in the *x*, *y*, and *z* directions for each cluster [41]:

(2) $$R_{g}=\sqrt{R_{gx}^{2}+R_{gy}^{2}+R_{gz}^{2}}$$

The nanocluster number density was determined by:

(3) $$N_{nc}=\frac{N_{C}}{V_{T}}$$

where *V_T_* is the analyzed volume of a given needle, and *N_C_* is the number of clusters identified using the maximum separation method.

## 3. Results

### 3.1. As-Received and Proton-Irradiated

**Figure 1:**
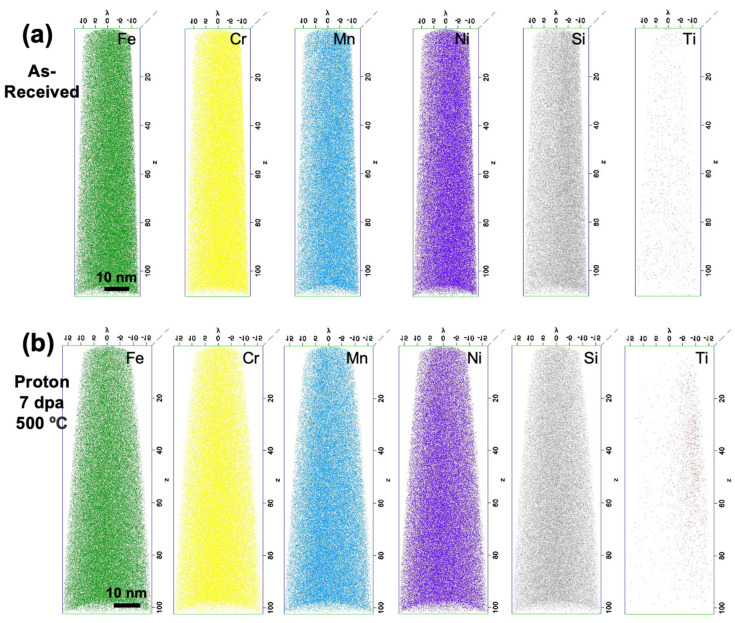
Representative APT needle reconstructions for (**a**) as-received and (**b**) proton-irradiated D9, showing absence of clustering of all major alloying species Fe, Cr, Mn, Ni, Si, and Ti.

Three APT needles were reconstructed from the as-received material, and five from the proton-irradiated specimen. All of the reconstructions show homogeneous distributions of all major alloying elements, Fe, Cr, Mn, Ni, and Si, in both the as-received and proton-irradiated specimens, as shown in Figure 1. This degree of homogeneity is expected in the as-received specimen, since previous characterization studies of this heat of D9 [26] show no evidence of pre-existing nanoclusters. The Ti distribution is also homogeneous in the as-received specimen but appears concentrated toward a side of the proton-irradiated specimen. This skewing is likely an artifact of the Ti peak in the APT mass spectrum (Da~45) being significantly larger than that of the other elements studied; APT laser settings optimized for elements such as Fe, Cr, and Ni, which have peaks in the range Da ~20–30, are not as ideal for the larger Da value of Ti.

The proton-irradiated needles reveal no evidence of irradiation-induced nanoclustering or nanoprecipitation. It is also worth noting that no chemical segregation to features such as grain boundaries, dislocation lines, or dislocation loops is observed in any of the as-received or proton-irradiated needles. However, the APT needle preparation process did not specifically aim to capture grain boundaries. Additionally, the likelihood of capturing dislocation loops or lines in the nanoscopic volume of an APT needle is low, and such features can also pop out to the surface of needles upon application of the APT laser pulse. Hence, no conclusive statements about nano-segregation behaviors can be drawn.

### 3.2. Neutron-Irradiated

**Figure 2:**
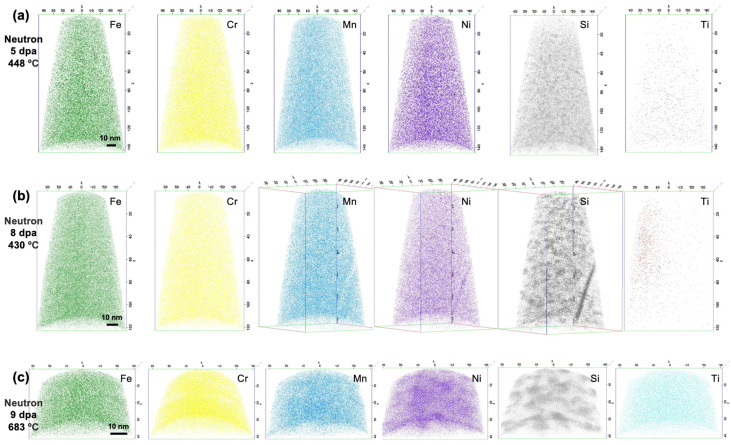
Representative APT needle reconstructions for neutron-irradiated D9 showing Ni and Si nanoclustering at (**a**) 5 dpa, 448 °C, (**b**) 8 dpa, 430 °C, and (**c**) 9 dpa, 683 °C; all other major alloying species (Fe, Cr, Mn, Ti) exhibit no evidence of clustering.

A total of six, two, and five APT needles were reconstructed from the 5, 8, and 9 dpa neutron-irradiated specimens, respectively, with representative reconstructions shown in Figure 2. At all neutron irradiation conditions, a fine dispersion of Ni–Si rich nanoclusters forms throughout the material. The other alloying elements of Fe, Cr, Mn, and Ti are not enriched in the nanoclusters and are homogeneously distributed, although the Ti distribution appears skewed for the 8 dpa condition, likely due to the same mass spectrum artifact noted in the proton-irradiated specimen. The average Ni concentration in nanoclusters increases with irradiation dose, from 22.53 to 32.74 at%, as shown in Table 4. The Si concentration in nanoclusters also increases with irradiation dose, maintaining a statistically invariant Ni/Si ratio in nanoclusters ranging from 3.08–3.25.

**Figure 3:**
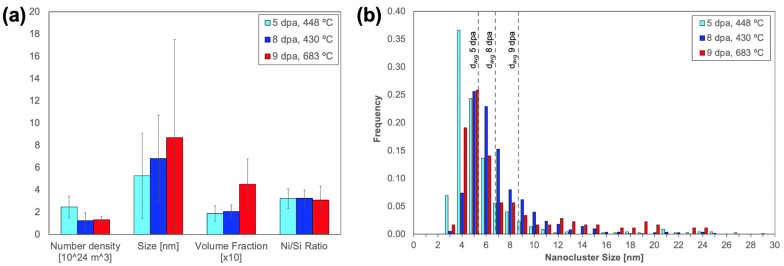
Comparison of (**a**) nanocluster morphologies and compositions and (**b**) nanocluster size distributions across neutron irradiation conditions.

The average nanocluster morphologies are also summarized in Table 4 and presented in Figure 3a. The average nanocluster size increases from 5.3 ± 3.8 nm at 5 dpa, through to 8.7 ± 8.8 nm at 9 dpa. Meanwhile, the nanocluster number density exhibits a statistically significant decrease with increasing dose, from 2.45 ± 0.99 × 10^24^ m^−3^ at 5 dpa, through to 1.31 ± 0.31 × 10^24^ m^−3^. This coarsening behavior is reflected by an increase in nanocluster volume fraction, which is 0.19, 0.21, and 0.45, at 5, 8, and 9 dpa, respectively. The volume fraction increase is only statistically significant between the 8 dpa, 430 °C and the 9 dpa, 683 °C conditions, suggesting that volume fraction increase may be most strongly influenced by temperature. The nanocluster size distribution, as shown in Figure 3b, is positively skewed at all doses. However, the magnitude of skewness decreases with increasing dose, which is also consistent with nanocluster coarsening.

**Figure 4:**
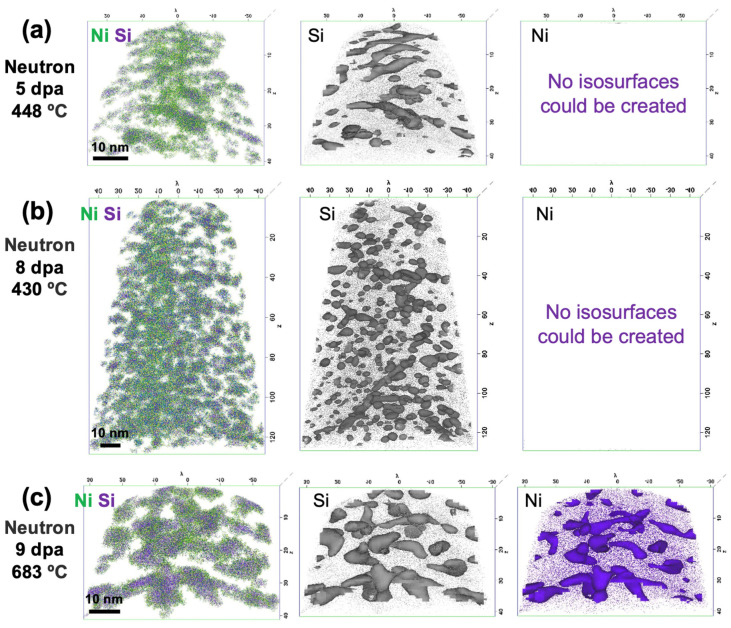
Nanoclusters in (**a**) 5 dpa, 448 °C, (**b**) 8 dpa, 430 °C, and (**c**) 9 dpa, 683 °C D9, showing for each irradiation condition: the Ni and Si distributions in nanoclusters, 10 at% Si isosurfaces, and 30 at% Ni isosurfaces.

Nanoclusters appear to have a core–shell type structure in which the Ni-rich core is surrounded by a Si-rich shell, as shown in Figure 4. While this core–shell structure seems to appear at all irradiation conditions (Figure 4a,b), it is most clearly observed in the 9 dpa, 683 °C condition, as shown in Figure 4c. Isoconcentration surfaces are defined for concentrations near the average nanocluster concentration of 10 at% Si and 30 at% Ni (i.e., indicative of the Ni_3_Si stoichiometry). At the lower irradiation temperatures, Ni isoconcentration surfaces cannot be defined, confirming that the nanoclusters do not achieve Ni_3_Si stoichiometry. At the higher irradiation temperature, the isoconcentration surfaces for Ni are smaller than for Si, as shown in Figure 4c, which is further indicative of the core–shell structure. The nanoclusters are more complex in shape and morphology at the 9 dpa, 683 °C condition than at the lower doses and temperatures; the isosurfaces appear nearly interconnected like spinodal-type phases.

### 3.3. Radiation Induced Segregation

**Figure 5:**
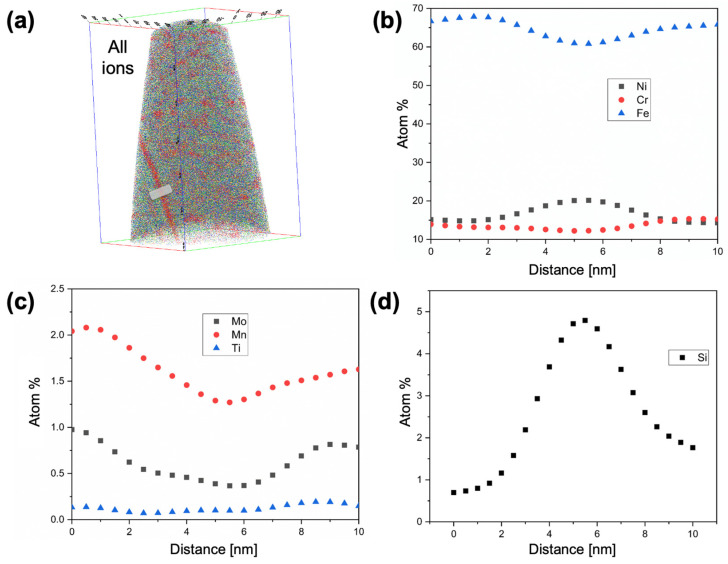
Example of grain boundary RIS in 8 dpa, 430 °C D9, showing (**a**) APT needle reconstruction containing all ranged ions and marked region over which composition line scan is taken to obtain RIS profiles of (**b**) major alloying elements Fe, Ni, and Cr, (**c**) Mo, Mn, and Ti, and (**d**) Si.

One APT needle from the 8 dpa, 430 °C neutron-irradiated specimen passed over a grain boundary. The RIS behavior across this boundary is summarized in Figure 5. The boundary is depleted of Fe, Cr, Mo, and Mn; limited depletion of Ti also occurs. The only two species that enrich at the grain boundary are Ni and Si, consistent with the species that form into irradiation-induced nanoclusters. The Ni/Si ratio at the grain boundary is ~4.3, but little can be inferred from this composition ratio, since the as-received grain boundary chemistry has not been captured in any of the APT needles. It is also worth noting that grain boundary RIS tends to be sensitive to grain boundary misorientation angle [42,43], and that grain boundary segregation patterns are also sensitive to the orientation and associated stress fields surrounding dislocations that comprise grain boundaries [44].

## 4. Discussion

Irradiation-induced Ni–Si nanoclusters or γ’-phase nanoprecipitates have long been known to form in austenitic stainless steels [4,7,26,45,46,47,48,49,50,51,52,53,54]. Zinkle and colleagues’ 1993 compilation [55] of irradiation-induced precipitation behaviors in legacy specimens of solution-annealed 316 SS suggests that the γ’ precipitate phase forms within a limited range of temperatures, from 400–500 °C, and doses exceeding ~40 dpa. They also note that fine γ’ precipitates can form at higher temperatures after only a few dpa, but subsequently dissolve at higher doses. Table 5 summarizes a more comprehensive set of observations of irradiation-induced Ni–Si nanoclusters from the archival literature in a wider range of commercial and model austenitic stainless steels; the cluster sizes, number densities, compositions, and irradiation conditions are tabulated, if reported. Most notably, this summary of Ni–Si nanoclusters suggests that Ni–Si nanoclusters and γ’ precipitates can exist over a considerably wider irradiation temperature and dose envelope than previously suggested. That is, there is evidence of Ni–Si nanoclustering in 304 SS, 316 SS, D9, and a model austenitic steel at temperatures ranging from 200–683 °C and doses ranging from 2–80 dpa.

**Figure 6:**
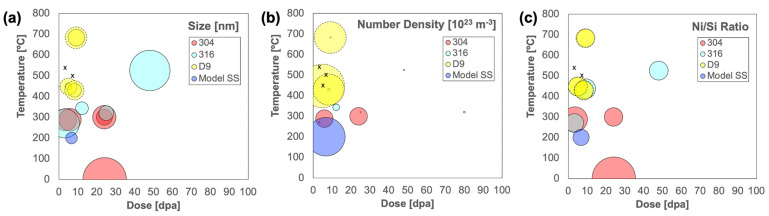
Bubble plots illustrating the relative (**a**) size, (**b**) number density, and (**c**) Ni/Si ratio of irradiation-induced Ni–Si nanoclusters, nanoprecipitates, and γ’ phases measured in D9 and other austenitic stainless steels in this study (dashed outlines) and from the archival literature (solid lines), specifically refs. [4,7,26,45,46,47,48,49,50,51,52,53,54]; the size of bubble indicates relative size of the parameter being plotted, and the “x” symbols indicate conditions in which no Ni/Si nanoclusters are observed.

Although the summarized data are limited and were collected over nonsystematic irradiation conditions and from different process heats of alloys, a few general trends can be extracted. First, the size and number density evolution of nanoclusters may suggest coarsening with increasing dose and/or temperature; this can be seen visually with bubble plots, as shown in Figure 6a,b. Quantitative measurements of nanocluster sizes and densities in the present study are self-consistent and indicative of coarsening with dose and/or temperature. However, by comparison to other studies that use TEM, the use of APT herein resolves smaller nanoclusters at a higher number density. It is also worth noting that nanocluster number densities in 304 SS tend to be several orders of magnitude greater than those in 316 SS, although it is unclear whether this is due to actual differences in bulk composition or solute chemistry between the alloys, or whether this is merely an artifact of the limited data set available. The nanocluster sizes and densities appear insensitive to bulk Si concentration, although the summarized studies do not use consistent methods of measuring Si concentration.

At elevated doses, Ni–Si nanoclusters are generally considered to be γ’-phase precipitates, which have simple cubic structures with Ni_3_Si stoichiometry [54,56] and lattice parameters of 0.3505 nm [48]. The current study is consistent with nearly all other reports in archival literature, showing that nanoclusters have approximately Ni_3_Si stoichiometry (i.e., Ni/Si ratio~3), as shown in Table 5. The stoichiometry appears insensitive to irradiation dose and temperature, as shown in Figure 6c, and to the measurement technique (APT or TEM). One notable case in which the nanoclusters deviate from Ni_3_Si stoichiometry is the Fe-17Cr-12Ni-1Si model steel irradiated with 6.4 MeV Fe^3+^ ions at 200 °C, from Fukumoto et al. [53]. They characterize the nanoclusters at three depths ranging from 500 nm (corresponding to 2.0 dpa) through the damage peak at 1500 nm (corresponding to 6.7 dpa); over this span, the nanocluster Ni/Si ratio ranges from 2.1–2.5. This is the only study summarized in Table 5 that focuses on a high-purity model alloy and the only study in which ions induce Ni–Si nanocluster nucleation; these unique aspects may contribute to the deviation from the Ni_3_Si nanocluster stoichiometry.

The Ni–Si precipitation mechanism remains unclear. Historically, Ni–Si precipitates have been thought to initially nucleate at interstitial sinks [49,57], suggesting an interstitial diffusion-driven nucleation mechanism. With increasing fluence, Ni–Si precipitates nucleate in the matrix, due to RIS eventually causing compositional segregations at all defect sinks [55,58,59,60]. But RIS of Ni in austenitic stainless steels is ascribed to diffusion via the vacancy flux, rather than via the interstitial flux [61,62]. When combined, these theories might suggest that the transport of Ni to nanoclusters is at first interstitial-mediated but evolves to be vacancy-mediated. While this is plausible, it requires significant co-evolution of sink strengths of multiple microstructural features in the system. The possible transition from interstitial- to vacancy-mediated clustering could also be consistent with rate theory studies in pure Ni–Si that show that irradiation-induced γ’ growth has a lower activation enthalpy at irradiation temperatures > 590 °C than at temperatures ranging from 475–570 °C [63].

Alternatively, the Ni core–Si shell structure of the nanoclusters observed in the present work may be able to shed light on possible nucleation mechanisms. This core–shell structure has not been reported in other studies; although Toyama et al. [7] describe a Mn core–P shell type structure of Ni–Si rich precipitates in 304 SS fuel wrapper plates (24 dpa, 300 °C), Ni and Si do not themselves form a core–shell structure in that work. The Ni–Si core–shell structure is consistent with the evolution of nanoclusters during post-irradiation annealing as reported in refs. [47,53,54]. For example, Jiao et al. [47] conduct post-irradiation annealing of specimens from a 304L SS control rod (5.9 dpa, maximum temperature 288 °C during full insertion). While the as-irradiated Ni–Si nanoclusters do not exhibit a core–shell structure, Si diffuses more rapidly than Ni out of the clusters upon post-irradiation annealing [47], leaving a Ni core-like structure after partial annealing. Similarly, Fukumoto et al. [53] observe that the Ni/Si ratio of ion irradiation-induced nanoclusters increases with post-irradiation annealing temperature, suggesting that Si diffuses out of nanoclusters more rapidly than does Ni. Even grain boundary segregation of Ni and Si exhibit similar post-irradiation annealing phenomena, in which Si recovers more readily than Ni [54]. It is possible that similar phenomena may be occurring in the present study, in which Si may be diffusing out of nanocluster peripheries after irradiation, artificially creating the appearance of the Si core–Ni shell structure. It may also be plausible that these nanoclusters form this core–shell structure as a result of segregation to dislocation loops, as their number density is inconsistent with Chen and colleagues’ measurements of dislocation loop densities in the identical material [26].

In austenitic stainless steel or γ-Fe, Si [64] has a higher diffusion coefficient than Ni [65,66]. Additionally, the activation energy for Ni diffusion (via vacancies or interstitials) [67] is higher than that for Si diffusion [68]. Thus, since Ni is a slower diffuser than Si, a Ni core with Si shell structure suggests that nanocluster cores do not initially nucleate by diffusion. Rather, the Ni cores may form through local compositional fluctuations [69], which can be likened to spinodal decomposition; this can also explain the interconnected and spinodal-like appearance of the isosurfaces (Figure 4b,c). A similar nucleation mechanism has been reported in U-50 wt% Zr under irradiation, in which a spinodal-like decomposition evolves into binodal precipitate growth [70,71]. Consequently, a modified U–Zr phase diagram has been proposed with extended spinodal and binodal curves; similarly, the Fe–Ni phase diagram has spinodal and binodal curves at the FeNi_3_ stoichiometry, which makes the hypothesized mechanism plausible. The nanoclusters are known to be coherent with the matrix, and their growth is believed to be promoted by coherency strain [53]. This, too, is consistent with a possible spinodal-like nucleation. The coherency leads to a high sink strength at nanocluster-matrix interfaces, and subsequent rapid radiation-enhanced diffusion of Si will result in further growth of the nanoclusters. The key role of Ni in driving the Ni–Si precipitation is similar to the dominant role of Ni in initiating G-phase precipitation in reactor pressure vessel (RPV) steels [72].

Finally, it is also worth commenting on the absence of Ni–Si nanoclusters in the proton-irradiated specimen herein. This observation is consistent with previous studies showing that ion [73,74] and proton [69,75,76,77] irradiation cannot replicate neutron irradiation-induced nanoclustering of a’ phases (sometimes described as Cr-rich nanoclusters) and Ni–Mn–Si–P (i.e., G-phase) nanoclusters in bcc Fe–Cr alloys, Fe–Cr–Al alloys [78], and oxide dispersion strengthened (ODS) steels. The exact mechanisms for this behavior are unknown but have been theorized to be associated with the accelerated irradiation damage rate of protons and ions compared to neutrons. However, ions may be able to induce Ni–Si nanoclustering in austenitic steels, considering the observations of nanoclusters due to 6.4 MeV Fe^3+^ ion irradiation in a study from Fukumoto et al. [53]. Nevertheless, the implanted ions may alter precipitation behavior [79].

## 5. Conclusions

APT was used to investigate austenitic stainless steel D9 irradiated with neutrons to 5 dpa at 448 °C, 8 dpa at 430 °C, and 9 dpa at 683 °C, and with 2 MeV protons to 7.0 dpa at 500 °C. Irradiation-induced nucleation of Ni–Si nanoclusters are consistently observed in all neutron-irradiated specimens, but they do not nucleate under proton irradiation. The nanoclusters coarsen with increasing neutron irradiation dose and temperature, but the Ni/Si ratio remains relatively constant near Ni_3_Si (i.e., γ’-phase) stoichiometry. The stability of these nanoclusters at the highest irradiation temperature, 682.5 °C, suggests that Ni–Si nanoclusters may be able to exist over a wider irradiation temperature range than previously thought. The observed nanoclusters exhibit a core–shell structure with a Ni-rich core and a more diffuse Si shell. Although a core–shell structure has not previously been reported for Ni–Si nanoclusters, it is consistent with post-irradiation annealing in the archival literature, which shows Si recovers more readily than Ni from nanoclusters. Because Ni is a slower diffuser than Si, the core–shell structure suggests that the Ni cores may nucleate through local compositional fluctuations akin to a spinodal-like phase instability, then subsequently grow through rapid Si diffusion to the core–matrix interface.

## Figures and Tables

**Table 1:** Alloy composition (wt%).

**Method**	**Fe**	**Ni**	**Cr**	**Mo**	**Mn**	**Si**
Nominal [25]	Bal.	16.27	13.35	1.55	1.70	–
EDS [26]	Bal.	17.3 ± 0.3	12.86 ± 0.07	1.27 ± 0.06	1.87 ± 0.03	1.25 ± 0.05
APT	65.6	15.2	13.95	2.20	2.03	0.66
**Method**	**Ti**	**Nb**	**V**	**Al**	**Cu**	**Co**
Nominal [25]	–	–	0.02	0.02	0.01	–
EDS [26]	0.11 ± 0.03	0.01 ± 0.01	–	0.09 ± 0.02	–	–
APT	0.26	0.02	0.02	0.014	0.005	0.014

**Table 2:** Irradiation conditions studied.

Irradiating Particle	Dose [dpa]	Temperature [°C]
Fast neutrons	5	448 ± 20
Fast neutrons	8	430 ± 20
Fast neutrons	9	683 ± 20
2 MeV protons	7	498 ± 7.5

**Table 3:** Summary of APT needles from each specimen condition, including APT run parameters, cluster analysis parameters, and number of clusters identified in each needle.

Irradiation Condition	RHIT File Name	Laser Energy [NJ]	No. of Ions [million]	Pulse Rate [kHz]	Detection Rate [%]	Base Temp. [K]	*d_max_* [nm]	*N_min_* [ions]	No. of Clusters
As Received	07660	0.1	2	200	0.5	40.3	n/a	n/a	n/a
	07731	0.1	9.5	250	0.5	60.5	n/a	n/a	n/a
	07596	0.1	1.2	200	0.5	55	n/a	n/a	n/a
Neutron, 5 dpa, 448 °C	07273	0.08	5	200	0.5	49.7	0.56	34	141
	07274	0.08	1.5	200	0.5	49.7	0.54	31	39
	07279	0.1	2	200	1.0	49.7	0.58	39	69
	07597	0.1	2.2	200	0.5	55	0.60	32	70
	07662	0.1	6.5	200	0.5	39.5	0.60	41	129
	07598	0.14	50	200	1.0	55.1	0.60	40	271
Neutron, 8 dpa, 430 °C	07605	0.14	35	200	0.8	54.9	0.63	81	351
	07606	0.14	48	200	0.5	55	0.61	68	706
Neutron, 9 dpa, 683 °C	07267	0.07	2.25	200	0.5	56.1	0.49	27	66
	07269	0.1	9	200	0.5	48.5	0.52	29	59
	07270	0.1	1.3	200	0.7	49.5	0.53	22	20
	07271	0.1	1.5	200	0.5	49.6	0.41	25	18
	07272	0.08	8	200	0.5	49.6	0.50	22	15
Proton, 7 dpa, 500 °C	07055	0.1	6	200	0.5	49.7	n/a	n/a	n/a
	07056	0.1	5.5	200	0.5	49.7	n/a	n/a	n/a
	07057	0.1	6	200	0.5	49.6	n/a	n/a	n/a
	07058	0.06	0.8	200	0.5	49	n/a	n/a	n/a
	07068	0.07	3.75	200	0.5	49.7	n/a	n/a	n/a

**Table 4:** Average nanocluster morphologies and compositions (at%) determined from APT.

Irradiation	Number Density [10^24^ m^3^]	Size [nm]	Volume Fraction	Average Composition [at%]								
				Fe	Ni	Cr	Mo	Mn	Si	Ti	C	Ni/Si Ratio
5 dpa 448 °C	2.45 ± 0.99	5.3 ± 3.8	0.19 ± 0.07	56.58	22.53	11.36	0.60	1.48	7.00	0.22	0.03	3.22
8 dpa 430 °C	1.24 ± 0.71	6.8 ± 3.9	0.21 ± 0.06	52.84	25.52	10.91	0.84	1.45	7.85	0.28	0.02	3.25
9 dpa 683 °C	1.31 ± 0.31	8.7 ± 8.8	0.45 ± 0.23	45.71	32.74	8.61	0.48	1.04	10.64	0.47	0.02	3.08

**Table 5:** Summary of Ni–Si nanocluster, nanoprecipitate, and γ’ phases reported in the archival literature (“n.s.” = not specified, “n.o.” = precipitates not observed, “–” = precipitates present but value not reported).

Alloy	Si Conc. [wt%]	Irradiating Particle	Dose [dpa]	Temp. [°C]	Average Size [nm]	No. Density [10^24^ m^−3^]	Ni/Si Ratio	Technique	Ref.
304	0.57	n (EBR-II)	24.15	371–389	~35	–	16.5	TEM	[45]
304	0.45	n	3.5	288	~8–10	–	5.5	APT	[46]
304	0.30	n (Barsebäck)	5.9	288	9.2 ± 0.7	0.39 ± 0.06	–	APT	[47]
304	0.78	n (Chooz A)	24	300	4.7	–	–	TEM	[4]
304	0.78	n	24	300	10	0.4	3	TEM	[7]
316	0.53	n (Tihange)	80	320	–	0.005	–	TEM	[48]
316	0.72	n (Tihange)	12.2	343	3	0.06	–	TEM	[49]
316	n.s.	n	9.2	425–450	–	–	3.4	TEM	[50]
316	n.s.	n (Dounreay)	3.2	270–540	8–23	0.0008–0.0042	~3	TEM	[51]
316	n.s.	n (Dounreay)	3.2	≥540	n.o.	n.o.	n.o.	TEM	[51]
316	0.51	n (EBR-II)	48	525	30	~0.001–0.003	3.14	TEM	[52]
316	0.62	n	25	320	4	0.001	–	TEM	[54]
Fe-17Cr-12Ni-1Si	0.96	6.4 MeV Fe^3+^	2–6.7	200	2.5	1.8–2	2.1–2.5	APT	[53]
D9	0.66	Neutron	5	448	<1	–	3	TEM	[26]
D9	0.66	Neutron	8.2	430	3.5 ± 0.1	0.008 ± 0.003	3	TEM	[26]
D9	0.66	Neutron	9.2	683	5.1 ± 0.3	0.002 ± 0.001	<3	TEM	[26]
D9	0.66	Neutron	5	448	5.27 ± 3.81	2.45 ± 0.99	3.22 ± 0.87	APT	This study
D9	0.66	Neutron	8	430	6.82 ± 3.88	1.24 ± 0.71	3.25 ± 0.74	APT	This study
D9	0.66	Neutron	9	683	8.70 ± 8.81	1.31 ± 0.31	3.08 ± 1.24	APT	This study
D9	0.66	Proton	7	500	n.o.	n.o.	n.o.	APT	This study

## Data Availability

Data is available upon request to the corresponding authors.
